# Histological analysis of the effects of a static magnetic field on bone healing process in rat femurs

**DOI:** 10.1186/1746-160X-2-43

**Published:** 2006-11-24

**Authors:** Edela Puricelli, Lucienne M Ulbrich, Deise Ponzoni, João Julio da Cunha Filho

**Affiliations:** 1 Oral and Maxillofacial Surgery Unit, Hospital de Clinicas de P.A., School of Dentistry, UFRGS, Porto Alegre, RS, Brazil; 2 Dept. of Oral Maxillofacial Surgery, School of Dentistry, UFRGS, Porto Alegre, RS, Brazil

## Abstract

**Background:**

The aim of this study was to investigate, *in vivo*, the quality of bone healing under the effect of a static magnetic field, arranged inside the body.

**Methods:**

A metallic device was developed, consisting of two stainless steel washers attached to the bone structure with titanium screws. Twenty-one Wistar rats (*Rattus novergicus albinus*) were used in this randomized experimental study. Each experimental group had five rats, and two animals were included as control for each of the groups. A pair of metal device was attached to the left femur of each animal, lightly touching a surgically created bone cavity. In the experimental groups, washers were placed in that way that they allowed mutual attraction forces. In the control group, surgery was performed but washers, screws or instruments were not magnetized. The animals were sacrificed 15, 45 and 60 days later, and the samples were submitted to histological analysis.

**Results:**

On days 15 and 45 after the surgical procedure, bone healing was more effective in the experimental group as compared to control animals. Sixty days after the surgical procedure, marked bone neoformation was observed in the test group, suggesting the existence of continued magnetic stimulation during the experiment.

**Conclusion:**

The magnetic stainless steel device, buried in the bone, *in vivo*, resulted in increased efficiency of the experimental bone healing process.

## Background

Bone neoformation is of primary importance for the success of dental clinical-surgical treatments. Much attention has been given to the research of new strategies to improve oral maxillofacial surgical techniques, as well as on the knowledge and application of biomaterials [1] an their possible chemical and physical consequences on the patients.

Electromagnetic fields have been used for the stimulation of bone neoformation processes. Their effects are observed in the treatment of osteoporosis, osteonecrosis, osteotomized areas, integration of bone grafts and post-traumatic pseudarthrosis [2]. Several cell functions were also shown to be influenced by electromagnetic fields [3,4]. Electromagnetism affects osteogenesis through mechanisms such as neovascularization, collagen production, proliferation and differentiation of osteogenic cells, and the maintenance of the molecular structure of the extracellular matrix [5-7].

The objective of the present study is to contribute to the understanding of processes involved in the response of bone to electromagnetic fields, by evaluation of cortical and trabecular bone neoformation. Cell stimulation was induced by static, *in vivo *buried magnetic fields.

## Methods

Twenty-one male Wistar rats (*Rattus novergicus albinus*) were used in this randomized experimental study, aiming at the use of permanent magnetic fields buried *in vivo*. The animals were six-months old and weighed in average 450 grams. They were divided into three experimental and control groups, which were analyzed on days 15, 45 and 60 after beginning of the experiment.

The metal devices consisted of commercially pure martensitic stainless steel washers and titanium screws. The screws measured 1.0 mm in diameter, 0.5 mm in thread pitch and 2.0 mm in length. The pre-made magnetized washers were 3.0 mm in outer diameter, 1.5 mm in core diameter and 0.5 mm in thick. They were held over a 60 mm × 12 mm × 5 mm magnet during the sterilization process and surgery. The magnetic field was 41 Gauss (G). Calculations were performed at the Electromagnetism Laboratory, Physics Institute from Universidade Federal do Rio Grande do Sul.

The animals were anesthetized by intraperiotoneal injection of sodium tiopenthal in a dose of 25 mg/kg body weight and local infiltration of 3% prilocaine with felypressin.

**Figure 1:**
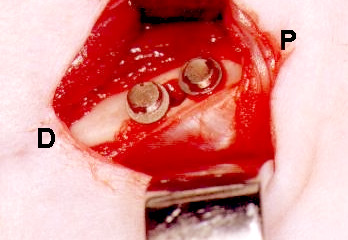
Distribution of screws and washers, outlining the borders of the surgical bone cavity. A distance of 1.3 mm separates the washers over the surgical cavity, corresponding to the area where the magnetic field operates. P and D mark, respectively, the proximal and distal regions of the left femur.

After reaching the medial portion of the left femur diaphisis, a surgical bone cavity was produced with a trephine (PROMM^®^, Comércio de Implantes Cirúrgicos Ltda. Porto Alegre, RS, Brazil) measuring 2.0 mm diameter active region, with low rotation and constant irrigation. Two holes were drilled with a drill guide (PROMM^®^) 1.0 mm away from the osteotomized border, one of them proximal and the other one distal to the surgical bone cavity. The washers were attached to the bone structure with titanium screws (Figure 1). A magnetic field was created in animals of the test groups, by placing up the north and south poles of the distal and proximal washers. In control animals, surgery included non-magnetized instruments, washers and screws.

The placement and stability of implants were confirmed by radiographic examination at the end of the experiments. Samples were submitted to longitudinal sectioning of the femur, which allowed simultaneous examination of the surgical cavity between the screw holes. The samples were prepared in hematoxylin and eosin stain (HE) for histological analysis.

## Results

**Figure 2:**
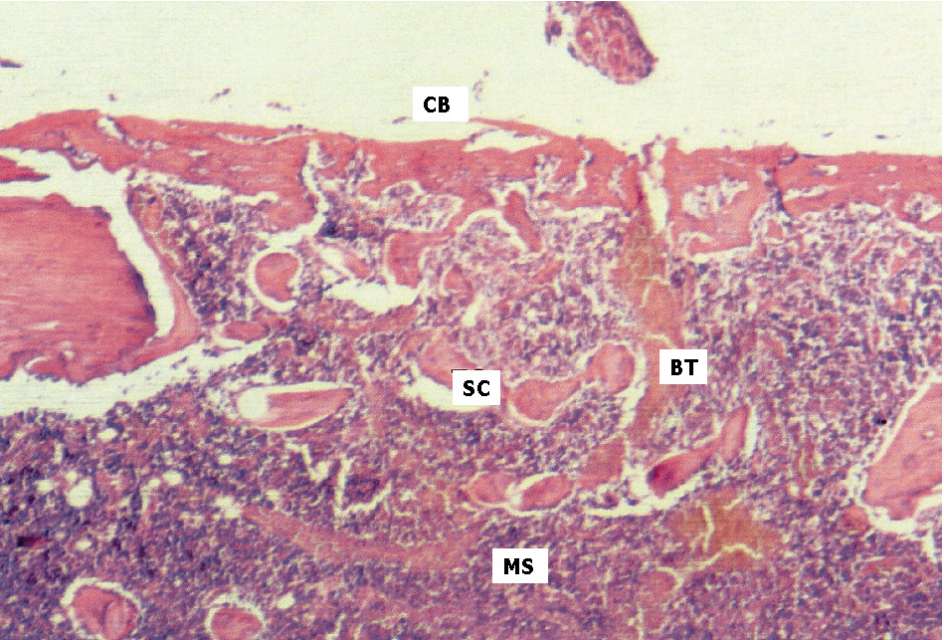
Control group, day 15. Surgical cavity (SC) limited on the upper side by cortical neoformation linearly continuous to borders (CB). Beginning of bone trabeculae in centripetal direction (BT). Medullary spaces showing connective tissue of great cellularity (MS). (HE 40×).

**Figure 3:**
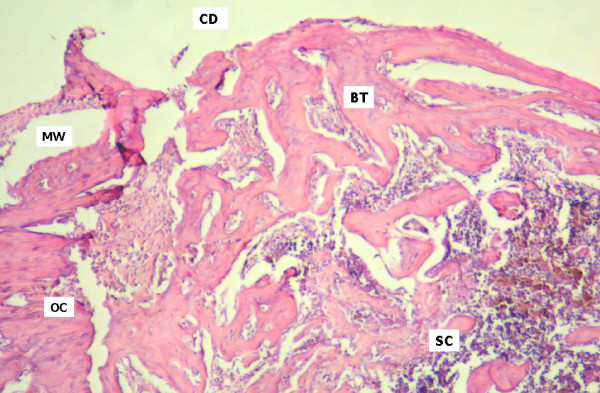
Test group, day 15. Surgical cavity (SC) with extensive centrifugal trabecular formation. Beginning of bone trabeculae in centrifugal direction (BT, CD). In (OC), osteotomized cortical bone marks the border of the cavity, supporting the magnetized washer (MW) (HE 40×).

On day 15, extensive trabecular formation with marked osteoblastic activity was seen on the cortical marginal zone of samples from animals of the control group, beginning in the endosteum close to the osteotomized cortical surface. On the external surface, its predominantly horizontal and flat direction maintained continuity and shape of the remaining cortical levels. Trabecular proliferation was also apparent in a centripetal direction relative to the surgical cavity. Medullary spaces showed connective tissue which was richly populated with cells and intense osteoblastic activity (Figure 2). In animals from the test group, trabecular formations presented a marginal centripetal direction relative to the magnetic field. The cortical wall on the osteotomized area presented a tendency to convexity, escaping from the horizontal outer border where the remaining cortical walls were located (Figure 3). Regular bone formation was observed following the limits of the magnetic washer. Medullary spaces were filled with numerous trabecular bone formations, showing a tendency for more abundant vertical growth. Rich hematopoietic tissue, with marked cell activity, could also be observed.

**Figure 4:**
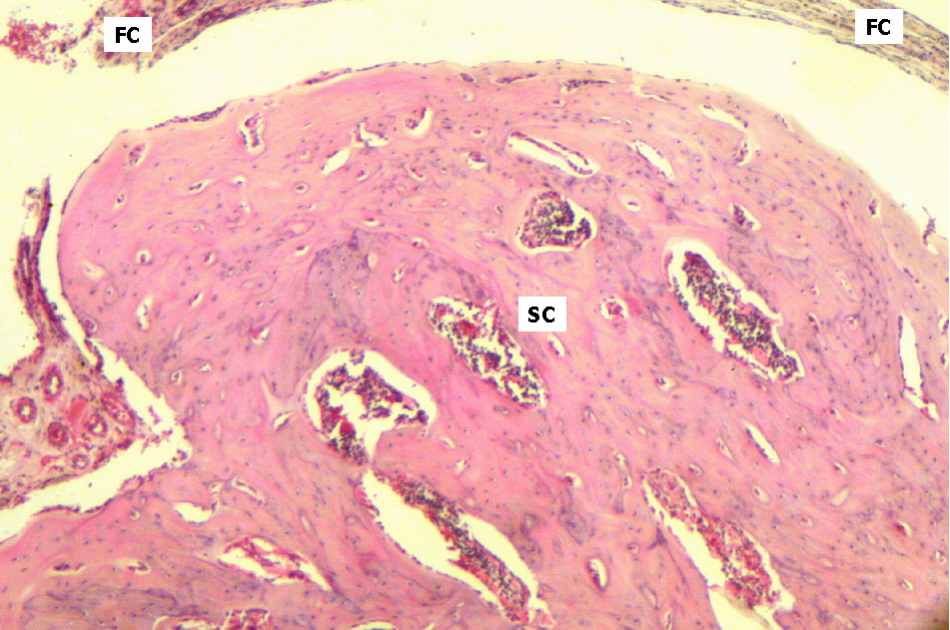
Control group, day 45. Surgical cavity (SC) with mature bone tissue, blood vessels and areas of internal remodelling. Fibrous capsules (FC) can be observed on the upper and lateral regions of the slide (HE 40×).

**Figure 5:**
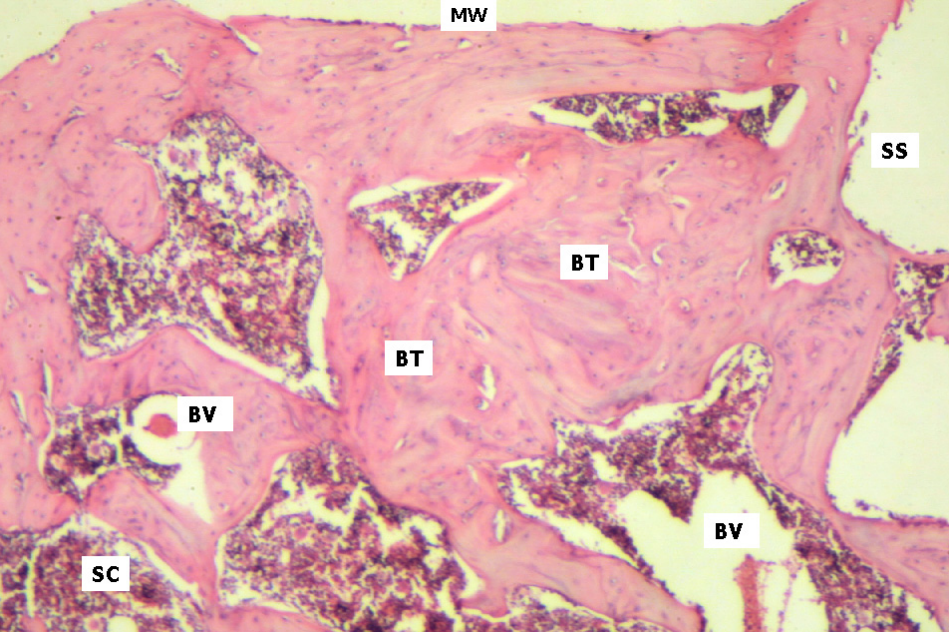
Test group, day 45. Surgical cavity (SC) with mature cortical and trabecular bone tissue characterized by lamellar structure. Blood vessels (BV) and areas of internal remodelling can be seen. Bone trabeculae remodeling (BT). Screw Space (SS). The neoformed area and the surrounding bone tissue (MW) show similar patterns (HE 40×).

On day 45, little activity was observed in the cortical zone of samples derived from the control group, which maintained convexity and showed predominant lamellar deposition. Areas limited by the washers showed fibrous tissue associated to osteoclastic activity. Medullary space was extensively invaded by bone trabecules and vascularized hematopoietic tissue (Figure 4). In the test group, the bone structure showed well organized areas of trabecular bone interspersed with medullary tissue. Blood vessels, adipose degeneration and osteoclastic activity were observed in medullary spaces, suggesting bone remodelling (Figure 5).

**Figure 6:**
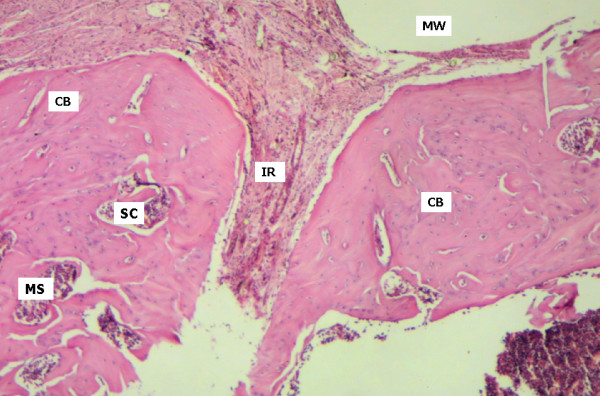
Control group, day 60. Surgical cavity (SC) covered by neoformed cortical bone in remodelling with similar process in the femur residual cortical. Inflammatory response to a foreign body (IR) is apparent. Medullary spaces (MS). Cortical Bone (CB). Space corresponding to the washer (MW) (HE 40×).

**Figure 7:**
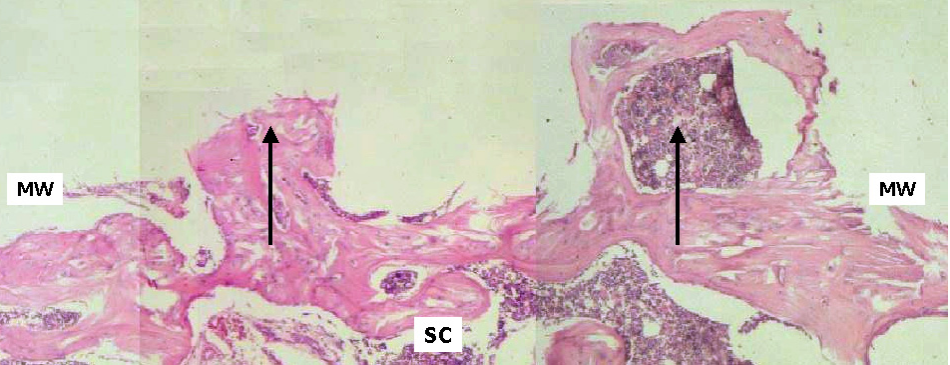
Test group, day 60. Photograph showing the surgical cavity sequence (SC). Centrifugal growth (↑), limiting the space corresponding to the magnetized washers are observed (MW). Bone remodelling with normal histological patterns, going beyond the cortical external border, is observed (HE 40×).

On day 60, active remodelling of the surgical cavity was apparent in samples from the control group, with normal cortical bone structures, trabecular spaces and hematopoietic tissue. Important thickening of the fibrous connective tissue was observed. This fibrous capsule is possibly due to an inflammatory reaction to the foreign body represented by the buried metallic device (Figure 6). In specimens from the test group, centrifugal growth, approximately symmetrical and bilateral in relation to the wound borders and reproducing the washers layout, was seen (Figure 7). Bone formation, surpassing the cortical level, showed recovery with normal characteristics. Distinct alterations were no longer present when the original and healed bone were compared, at the level of the medullary channel.

## Discussion

As in many other studies reported, rat was also used as a model in this study [1,6,8-10]. The advantages include easy manipulation, maintenance and adaptation to the objectives of the study. Other animals have been used, such as rabbits [7,11,12] or dogs [13].

This experimental study was based on investigations reported by Brighton (apud Christian) [14]; Burkitt, Young and Heath [15]; Hunter (apud Christian) [14]; and Lane and Davis (apud Christian) [14]. The surgically prepared bone cavity presented only one ruptured cortical, maintaining thus the reproducibility of a fixed fracture [16].

The metallic washers were attached to the bone structure with titanium screws. Biocompatibility of titanium with the spongeous medullary area has already been shown by Veeck, Puricelli and Souza [1]. Due to technical difficulties, the stainless steel washers were not protected against corrosion, differing thus from those used by Lemons and Natiella [17]. Martensitic stainless steel relates to the classification described by Chiaverini [18]. The need for externally adapted electric currents was avoided by the generation of a magnetic field through buried magnets, which resulted in a constant field with no need for reactivation during the experimental period.

A 41 G magnetic field was used, significantly higher than that of previously reported studies such as those of Grace, Revell and Brookes [5]; Matsumoto et al. [7]; Fini et al. [11]; Aaron, Wang and Ciombor [9]; and Ciombor et al. [10], in which intensities of 12 G, 2 G, 16 G, 16 G and 16 G were employed respectively. The expressive difference in charge was due to lack of calibration information in literature reports, and to the novelty represented by devices which keep an active, isolated field with no possible reactivation.

Different *in vitro *and *in vivo *experimental systems have been used for the investigation of electric fields effects in vital tissues. Bodamyali et al. [19] and Ishisaka et al. [3] described the use of weak magnets for *in vitro *cell stimulation, but observed little activity in this system. *In vivo *studies were performed by Grace, Revell and Brookes [6]; Matsumoto et al. [7]; Fini et al. [11]; Aaron, Wang and Ciombor [9]; Ciombor et al. [10] and Inoue et al. [13], with daily application of electromagnetic fields during 2, 8, 6, 1, 8 and 8 hours respectively. Experiments were conducted during periods between 2 days and 8 weeks, and the studies were characterized by the use of an electromagnetic field with continuous stimulation.

According to Halliday et al. [20], the electric neutrality of a body is modified when it is submitted to a magnetic field. Reports by Oishi and Onesti [2] and Teló [4] suggest that cell electronegativity at bone fractures and after cancer treatment should be regarded as a possible indication of electric modifications on the local wound.

The extensive trabecular formation beginning in the endosteum, histologically observed in the surgical bone cavity in samples from the test groups as early as 15 days later, suggests that the magnetic field stimulates bone healing.

On day 45, neoformed bone was rather similar to the surrounding bone tissue in test and control groups, showing the presence of a first intention healing process as stated by Lane and Danis (apud Christian) [14]. In the test group, however, stronger neovascularization as well as osteoclastic and bone remodelling activities were observed.

On day 60, besides marked external configuration of the magnetic washers with cortical bone, the establishment of bone projections beyond the external border of the previously osteotomized cortical was observed. These results suggest that the magnetic field was active during all the experimental period. Even though they cannot be strictly compared to the studies of Grace, Revell and Brookes [6]; Matsumoto et al. [7]; Fini et al. [11]; and Fredericks et al. [12], since these authors used intermittent electromagnetic fields, the results of the present work agree with the accelerated bone neoformation reported.

The histological observation of hematopoietic activity in the bone marrow is an important result. Urist, Delange and Finermann [21] and Grace, Revell and Brookes [6] suggested that cartilage formation is due to a shortage of blood supply. The results of the present study, with *in vivo *observations during a period of 60 days, show that blood supply to the region was not impaired, but on the contrary was stimulated, which may explain the absence of cartilage formation during the healing process.

The results of the present experimental work indicate that further studies are needed for the detailed analysis of the *in vivo *activity and best intensity of magnetic stimulation on healing bone tissue.

## Conclusion

The experimental approach used in this study allows the following conclusions:

1. The magnetized stainless steel material used in these studies is able to affect the bone healing process;

2. The comparison of test and control groups indicates that bone healing was accelerated by the effect of magnetic fields in all the conditions analyzed;

3. The marked configuration of a bone outline involving the metallic devices in the test group, observed until the end of the experimental period, suggests that the magnetic field exerted a constant local activity on the surgical wound.

## Competing interests

The author(s) declare that they have no competing interests.

## Authors' contributions

EP suggested the original idea for the study; initiated the investigations leading to these results; wrote the protocols for the study and for the Research and Ethics in Health Committee; participated in discussions on the undertaking of the study; conceived, designed, and supervised the study; interpreted the data; reviewed all iterations of the paper. LMU developed the dissertation on which this work is based; participated in discussions on the undertaking of the study, supervised and participated in obtaining the results, interpreted the data, reviewed the paper for content, and reviewed and contributed to the writing of all iterations of the paper. DP collaborated with laboratory experimental procedures and observation of animal bioethics guidelines; reviewed and contributed to the writing of all iterations of the paper, including the final version of the manuscript. JJCF participated in the analysis of results and implementation of material and other conditions for development of the project. All authors approved the final report.
